# Imported Malaria Outbreak in Military Personnel Returning from Peacekeeping Operation in South Sudan — Thailand, 2023

**DOI:** 10.4269/ajtmh.24-0735

**Published:** 2025-07-10

**Authors:** Anupong Sirirungreung, Parichat Sangwanborirak, Kasinpiti Naprasith, Kanlaya Jongcherdchootrakul, Porruthai Kittikanara, Watcharee Yokanit, Sutchana Tabprasit, Adisorn Lumpaopong, Ram Rangsin, Mathirut Mungthin

**Affiliations:** ^1^ Department of Military and Community Medicine, Phramongkutklao College of Medicine, Bangkok, Thailand;; ^2^ Health Promotion and Preventive Medicine Division, Royal Thai Army Medical Department, Bangkok, Thailand;; ^3^ Armed Forces Research Institute of Medical Sciences, Bangkok, Thailand;; ^4^ Phramongkutklao College of Medicine, Bangkok, Thailand;; ^5^ Department of Parasitology, Phramongkutklao College of Medicine, Bangkok, Thailand

## Abstract

Malaria remains a global health concern, with Thailand primarily experiencing cases in border areas and from imported infections. During the third rotation of Thai peacekeeping troops returning from South Sudan between June and November 2023, a malaria outbreak was reported. In response to the outbreak, we conducted a retrospective cohort study, in which we identified 46 confirmed cases of malaria, with 27 occurring postdeployment. The most common species were *Plasmodium falciparum* (*n* = 19) and *Plasmodium ovale* (*n* = 16), with a median postdeployment time to first symptom of 14 weeks (interquartile range: 9–28). The analysis revealed that those relying on informal sources for malaria knowledge, such as friends, were at higher risk (hazard ratio: 4.92; 95% CI: 1.97–12.28). Challenges with prophylaxis adherence and glucose-6-phosphate dehydrogenase deficiency-related hemolysis were identified. This study calls for improved malaria prevention in South Sudan, better postdeployment monitoring, enhanced education for troops, and research on terminal prophylaxis to reduce cases.

## INTRODUCTION

Malaria remains one of the most burdensome infectious diseases worldwide.^1^^,^^2^ In Thailand, the incidence of malaria has steadily decreased over the years; however, local transmission continues in villages near forested borders, and the frequency of imported cases is rising.^3^^–^^8^ *Plasmodium vivax* (*P. vivax*) is the most common species, followed by *Plasmodium falciparum* (*P. falciparum*), whereas *Plasmodium ovale* (*P. ovale*) is rarely seen.^3^

In South Sudan, where *P. falciparum* malaria is widespread and *P. vivax* and *P. ovale* are seldom reported,^9^^–^^11^ ongoing conflict and a complex humanitarian crisis have prompted the United Nations to call on member nations to deploy peacekeeping forces.^12^^–^^14^ Since 2021, Thailand has been among the countries that send 200–300 military personnel annually for peacekeeping operations in this region. Before deployment, these troops undergo a predeployment program that includes 2 weeks of training and preparation and covers malaria prevention strategies, such as mosquito bite prevention and malaria prophylaxis. The troops are provided with mosquito repellent creams or lotions and insecticide-treated nets for bite prevention. The malaria prophylaxis regimen consists of mefloquine (250 mg), which is taken weekly during the mission and for 4 weeks after returning.

Despite these measures, a few malaria cases were reported during and after the first and second rotations. Postdeployment screening for malaria infection was conducted by using thick blood smears and real-time polymerase chain reaction. However, between June and November 2023, after the military personnel returned from the mission, 31 malaria cases were identified among the third rotation of troops (*N* = 273). Initial reports revealed that nearly 30% of these cases were due to *P. ovale*, a species that is rarely found in Thailand, where more than 95% of cases are typically caused by *P. vivax* and *P. falciparum*, and *P. ovale* accounts for less than 1% of cases.^3^ An outbreak investigation was initiated to improve surveillance, understand case distribution, identify risk factors, and confirm malaria species.

## MATERIALS AND METHODS

We conducted a retrospective cohort study among Royal Thai Army personnel who returned from the United Nations mission in South Sudan (UNMISS), focusing on the third rotation, which occurred from April 2022 to June 2023. Active case finding and interviews using a structured questionnaire were performed between November 2023 and February 2024, with a 72% response rate (196/273), as troops returned to their home units across five provinces in Thailand, including Ratchaburi, Nakhon Ratchasima, Ubon Ratchathani, Udon Thani, and Roi Et. A flowchart of the study population is provided in Supplemental Figure 1.

A confirmed case was defined as an individual with malaria symptoms and positive results obtained via microscopy, rapid diagnostic tests, or quantitative polymerase chain reaction (qPCR) using the VIASURE Malaria differentiation Real Time PCR Detection Kit (CerTest Biotec, Zaragoza, Spain).^15^^,^^16^ This multiplex qPCR assay is used to detect and differentiate *P. falciparum*, *P. vivax*, *P. ovale*, *Plasmodium malariae*, and *Plasmodium knowlesi* by amplifying conserved regions of the 18S ribosomal ribonucleic acid gene using species-specific primers and fluorescent probes, based on the 5′ exonuclease activity of DNA polymerase.^16^ Upon arrival at the airport, all cohort members were tested by using thick film microscopy and qPCR by the Armed Forces Research Institute of Medical Sciences—Thai Component (AFRIMS). Those who developed malaria symptoms, such as fever, chills, headache, and muscle pain, after returning were identified through the surveillance system and tested for malaria at local hospitals.^17^^–^^19^ Blood specimens from these cases were then sent to AFRIMS for species confirmation using qPCR. The postdeployment time to first symptom was defined as the number of weeks (number of days divided by seven) from the arrival date to the first onset of symptoms, excluding those who tested positive during the arrival screening.

To identify risk behaviors and knowledge factors associated with postdeployment infection, an imported case was defined as a confirmed case in which symptoms developed after the arrival date, excluding those who reported symptoms or received positive laboratory results before arrival. Detailed information on the general characteristics of malaria cases during the deployment and postdeployment phases is presented in Supplemental Table 1. The incidence rate of imported malaria infection per person-week was calculated, starting from the arrival date in June 2023 (depending on the flight) and ending on March 1, 2024. Cox proportional hazard models were used to estimate hazard ratios (HRs), with potential confounders, including age (in years), income (in baht per year), and the province of the affiliated unit (Ratchaburi, Nakhon Ratchasima, and others), incorporated into the final models. The analyses were conducted using R with the “survival” package (version 3.5–8; R Foundation, Vienna, Austria) for the Cox proportional hazard model.

## RESULTS

The cohort characteristics are summarized in Table 1. The cohort was predominantly male (98%), with a median age of 36 years (interquartile range [IQR]: 32–41 years). Nearly half (48%) were stationed in Juba, 38% were stationed in Rumbek, and 14% were stationed in multiple areas, with all units regrouping in Juba before returning (June 12–26, 2023). After returning to Thailand, all participants reported living only in areas with no reported cases of infection for 3 consecutive years.^3^

**Table 1:** General characteristics of military personnel returning from peacekeeping operations in South Sudan—Thailand, 2023 (*N* = 196)*

Characteristic	*n*/Total (%)
Sex	
Female	4/196 (2)
Male	192/196 (98)
Age (years)	
25–29	23/196 (12)
30–34	68/196 (35)
35–39	44/196 (22)
40–44	30/196 (15)
45 and over	31/196 (16)
Median age (IQR)	36 (32–41)
BMI (kg/m^2^)	
Underweight (<18.5)	3/196 (2)
Normal (18.5 to <25)	119/196 (61)
Overweight (25 to <30)	60/196 (31)
Obesity (30 and over)	14/196 (7)
Median BMI (IQR)	24.3 (22.5–26.2)
Family income per year (baht)	
Less than 240,000	43/193 (22)
240,000 to <300,000	36/193 (19)
300,000 to <400,000	59/193 (31)
400,000 and over	55/193 (28)
Unknown	3
Median family income per year (IQR)	300,000 (240,000–400,000)
Province of affiliated unit	
Ratchaburi	128/196 (65)
Nakhon Ratchasima	53/196 (27)
Others	15/196 (8)
Main stationed city during the UNMISS	
Juba	95/196 (48)
Rumbek	74/196 (38)
Multiple cities	27/196 (14)
Continuous alcohol drinking (yes)	118/194 (61)
Unknown	2
Smoking status	
Current	71/195 (36)
Former	14/195 (7)
Never	110/195 (56)
Unknown	1

BMI = body mass index; IQR = interquartile range; UNMISS = United Nations Mission in South Sudan.

* Seventy-nine individuals were excluded due to unavailability for interviews.

Among the 273 troops, 46 malaria cases (17%) were reported. After excluding cases with missing important dates (*n* = 4), 6% (15/269) developed symptoms during deployment, and 10% (27/269) developed symptoms postdeployment, with an incidence rate of 4.3 per 1,000 person-weeks (95% CI: 2.9–6.3). Interviews conducted with 42 patients revealed that all were male, with a median age of 35 years (IQR: 33–39). Regarding deployment locations, 52% were stationed in Juba, 12% were stationed in Rumbek, and 36% were stationed across multiple cities (Supplemental Table 2).

**Figure 1.**
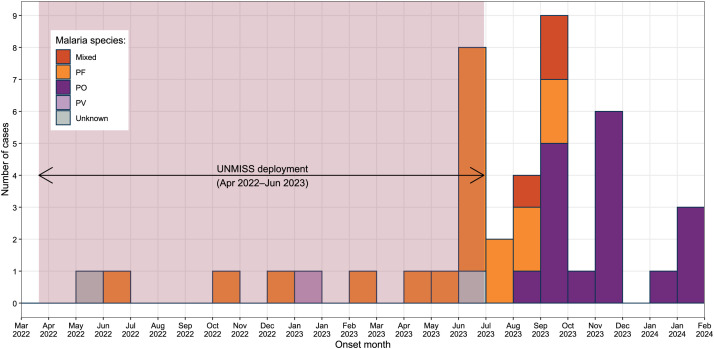
Number of malaria cases detected in military personnel returning from peacekeeping operations in South Sudan, Thailand, in 2023 (*n* = 46). *PF* = *Plasmodium falciparum*; *PO* = *Plasmodium ovale; PV = Plasmodium vivax;* UNMISS = United Nations Mission in South Sudan.

The epidemic curve by malaria species is shown in Figure 1. The highest number of *P. falciparum* cases occurred during the final weeks of the mission and in the first month after arrival, followed by a peak in *P. ovale* cases at ∼4 months postdeployment. In total, 19 *P. falciparum* cases, 17 *P. ovale* cases, one *P. vivax* case, and three mixed infections (*P. falciparum* and *P. ovale*) were identified, whereas six cases, including four without important date information, lacked species identification. The median postdeployment time to first symptom was 14 weeks (IQR: 9–28), with *P. falciparum* exhibiting a time to first symptom of 8 weeks (IQR: 3–11), *P. ovale* exhibiting a time of 16 weeks (IQR: 12–20), and the longest time to first symptom being 32 weeks.

The factors associated with the risk of malaria infection after returning from UNMISS in this cohort are highlighted in Table 2. The results indicate that those who cited friends as their primary source of malaria knowledge exhibited a higher risk of infection after the mission (HR: 4.92; 95% CI: 1.97–12.28). Other factors, including smoking and alcohol use, appeared to increase risk, although the CIs were wide.

**Table 2:** Incidence rates and hazard ratios for identified risk factors of malaria infection in military personnel returning from peacekeeping operations in South Sudan — Thailand, 2023 (*N* = 196)*

Risk Factor	Expose	Unexposed	Crude HR	Adjusted HR^‡^
	No. (IR)^†^	No. (IR)^†^	(95% CI)	(95% CI)
Source of malaria knowledge				
Internet (yes versus no)	13 (6.54)	14 (3.32)	1.93 (0.91–4.11)	1.93 (0.87–4.32)
Friends (yes versus no)	8 (13.85)	19 (3.37)	3.99 (1.74–9.13)	4.92 (1.97–12.28)
Major mission type				
Security (yes versus no)	5 (8.33)	22 (3.92)	2.13 (0.81–5.63)	1.19 (0.35–4.11)
Patrol (yes versus no)	8 (7.12)	19 (3.74)	1.86 (0.81–4.25)	1.20 (0.39–3.72)
Behavioral factors				
Continuous alcohol drinking (yes versus no)	21 (5.71)	6 (2.44)	2.34 (0.95–5.81)	2.01 (0.78–5.20)
Smoker (current or former versus never)	16 (6.26)	11 (3.04)	2.06 (0.96–4.45)	2.11 (0.97–4.61)
Obese (overweight or obesity versus normal)	14 (6.26)	13 (3.36)	1.82 (0.86–3.88)	1.98 (0.90–4.35)

HR = hazard ratio; IR = incidence rate; No. = number of events.

* Seventy-nine individuals were excluded because of unavailability for interviews.

^†^ Incidence rate per 1,000 person-weeks.

^‡^ Each adjusted HR was derived from an individual Cox proportional hazards model, accounting for the following covariates: age (years), income (baht per year), and province of affiliated unit (Ratchaburi, Nakhon Ratchasima, and others).

The results of our interviews on malaria prophylaxis adherence revealed that 92% of participants reported full prophylaxis predeployment, 97% reported full prophylaxis during deployment, and 89% adhered to the 4-week postdeployment mefloquine continuation. Side effects from mefloquine, including nightmares, insomnia, and visual hallucinations, affected 24% of participants, whereas 6% of participants reported nausea or vomiting.

All cases were successfully treated according to the national guidelines for the clinical management of malaria, primarily using dihydroartemisinin–piperaquine for *P. falciparum* and chloroquine and primaquine for *P. ovale* and *P. vivax*.^20^ Notably, four out of five individuals with confirmed glucose-6-phosphate dehydrogenase (G6PD) deficiency were diagnosed during malaria treatment. Hemolysis developed in three of these patients after primaquine administration, whereas one patient did not experience hemolysis complications. The affected individuals exhibited moderate hemolysis, which required the temporary cessation of primaquine and supportive management; all made a full recovery without lasting effects.

## DISCUSSION

Our investigation confirmed an imported malaria outbreak among military personnel returning from UNMISS, with the outbreak pattern suggesting a common source of exposure during a preflight gathering in Juba during the wet season.^21^^,^^22^ This rotation’s mission ended during the wet season, unlike previous dry-season rotations. Although imported *P. ovale* and *P. vivax* malaria from overseas missions in Africa have been reported previously,^23^^–^^28^ most malaria cases were caused by *P. falciparum*. This outbreak investigation underscores the ongoing risk and the need for improved prevention strategies, particularly for less recognized species like *P. ovale*.

We observed longer-than-usual times to first symptoms for both *P. falciparum* and *P. ovale*;^29^^,^^30^ however, these times were comparable to imported malaria cases in other countries.^31^^–^^34^ Several hypotheses may explain this finding. One is related to prophylaxis because longer incubation periods have been noted in populations taking malaria prophylaxis.^31^^,^^35^ The other concerns the mefloquine regimen, which is effective against blood-stage parasites but less so or ineffective against liver-stage hypnozoites, especially for *P. ovale.*^36^ In Thailand, the use of primaquine as terminal prophylaxis^37^ remains uncertain and excluded from the guideline^20^ because of G6PD deficiency risks, which affect 6–15% of the population.^38^^–^^41^

This investigation underscores the critical importance of routine G6PD deficiency screening before deploying personnel to malaria-endemic regions and before prescribing antimalarial regimens that involve primaquine.^42^ The occurrence of hemolysis, even when mild, reinforces the need to integrate G6PD testing into standard pretreatment protocols, especially in military and other high-risk populations.

In this study, high adherence to malaria prophylaxis was observed across all phases of deployment; however, nearly one-fourth of participants experienced neuropsychiatric side effects and gastrointestinal symptoms. Such adverse effects, particularly those caused by mefloquine, are known to affect compliance, especially postdeployment when supervision decreases.^43^^–^^46^ Reduced adherence may compromise prophylactic effectiveness and increase the risk of delayed-onset malaria, particularly that caused by *P. ovale* and *P. vivax*.^31^^,^^35^ These findings highlight the importance of predeployment counseling and the consideration of alternative regimens for those who are at risk of side effects.

The source of knowledge is crucial for effective malaria prevention among troops because it directly impacts their understanding and adherence to protective measures.^47^^–^^50^ Troops informed by medical professionals are better prepared than those who rely on informal sources (i.e., the internet and friends), underscoring the need for standardized education on malaria prevention both pre- and postdeployment.

The findings in this report are subject to a few limitations. First, we were unable to reach some target populations for interviews after their return, including four cases and 75 non-cases. Second, some information was gathered using structured questionnaires, and participants may have provided incomplete data. These factors could introduce selection and misclassification bias into the analysis of risk factors related to the interviews. However, the bias should be minimal for other results based on medical record reviews, such as time to first symptom and treatment.

Our assumption that the cases were imported is limited by the absence of molecular confirmation to verify the origin of the species.^51^^–^^54^ However, local malaria transmission among participants is highly unlikely. This conclusion is based on efforts to gather substantial evidence to reduce the likelihood of local transmission, including 1) accessing travel histories, which indicated that all soldiers lived in areas with minimal malaria transmission risk,^3^^,^^19^ and 2) knowledge of the fact that *P. ovale* infections are exceedingly rare, with only a few cases reported annually in the national malaria surveillance system.^3^ Thus, it is highly probable that the *P. ovale* outbreak resulted from imported cases. Although malaria screening was not performed before deployment, its absence likely had little effect on case detection because infections contracted before deployment would have become symptomatic during the 1-year mission. Nevertheless, malaria cases during deployment may be underreported because of less sensitive diagnostic methods in the field compared with the more advanced tools, including qPCR, available after return.

## CONCLUSION

Although no severe malaria cases were reported, the outbreak highlighted the need for the better preparation of troops for missions in malaria-endemic areas. Enhanced disease control measures are important, particularly during the postmission period when troops assemble before departure. Updated guidance includes G6PD screening to prevent hemolysis, active and extended post-deployment surveillance, and the awareness of delayed presentation with malaria after prophylaxis. It is crucial to improve predeployment malaria education, and further research on terminal prophylaxis is needed. Lastly, improving prevention strategies for *P. falciparum* and non-*P. falciparum* malaria should be considered. Incorporating advanced molecular analysis into the national surveillance system could further enhance the understanding of disease dynamics, particularly for imported malaria cases.

## Supplemental Materials

10.4269/ajtmh.24-0735 Supplemental Materials
