# Supplementary material for: Imported Malaria Outbreak in Military Personnel Returning from Peacekeeping Operation in South Sudan — Thailand, 2023
Source: Am J Trop Med Hyg. 2025 Jul 10;113(3):614–9. doi: 10.4269/ajtmh.24-0735 (PMC12410161; doi:10.4269/ajtmh.24-0735)
Supplement: Supplemental Materials [file tpmd240735.SD1.pdf]

Supplement Table 1. General characteristics of malaria cases during deployment and post-deployment phases among military personnel returning from peacekeeping operation in South Sudan — Thailand, 2023 (n=42)*

Characteristic	During deployment cases (%) n = 15	Post-deployment cases (%) n = 27
**Sex**		
Female	0 (0)	0 (0)
Male	15 (100)	27 (100)
**Age (years)**		
25–29	0 (0)	3 (11)
30–34	8 (53)	11 (41)
35–39	7 (47)	5 (19)
40–44	0 (0)	5 (19)
45 and over	0 (0)	3 (11)
Median age (IQR)	35 (33–37)	35 (31–41)
**BMI (kg/m^2)**		
Underweight (less than18.5)	0 (0)	0 (0)
Normal (18.5–<25)	7 (47)	13 (48)
Overweight (25–<30)	2 (13)	3 (11)
Obesity (30 and over)	6 (40)	11 (41)
Median BMI (IQR)	25.1 (24.1–26.6)	25.1 (23.1–27.1)
**Family income per year (baht)**		
Less than 240k	2 (14)	9 (35)
240k–<300k	4 (29)	1 (4)
300k–<400k	1 (7)	6 (23)
400k and over	7 (50)	10 (38)
Unknown	1	1
Median family income per year (IQR)	320,000 (296,160–	260,000 (200,000–

Characteristic	During deployment cases (%)<br>n = 15	Post-deployment cases (%)<br>n = 27
	400,000)	336,000)
**Province of affiliated unit**		
Ratchaburi	6 (40)	16 (59)
Nakhon Ratchasima	7 (47)	4 (15)
Others	2 (13)	7 (26)
**Main stationed city during the UNMISS**		
Juba	10 (67)	12 (44)
Rumbek	4 (27)	11 (41)
Multiple cities	1 (7)	4 (15)
**Continuous alcohol drinking (yes)**	11 (73)	21 (78)
**Smoking status**		
Current	11 (73)	13 (48)
Former	1 (7)	3 (11)
Never	3 (20)	11 (41)

Abbreviations: BMI = body mass index; UNMISS = United Nations Mission in South Sudan.
* Four cases were excluded due to unavailability for interviews.

Supplement Table 2. General characteristics of malaria cases and non-cases among military personnel returning from peacekeeping operation in South Sudan — Thailand, 2023 (n=196)*

**Characteristic**	**Cases (%)**<br>**(n = 42)**	**Non-cases (%)**<br>**(n = 154)**
**Sex**		
Female	0 (0)	4 (3)
Male	42 (100)	150 (97)
**Age (years)**		
25–29	3 (7)	20 (13)
30–34	19 (45)	49 (32)
35–39	12 (29)	32 (21)
40–44	5 (12)	25 (16)
45 and over	3 (7.1)	28 (18)
Median age (IQR)	35 (33–39)	36 (32–42)
**BMI (kg/m^2)**		
Underweight (less than18.5)	0 (0)	3 (2)
Normal (18.5–<25)	20 (48)	99 (64)
Overweight (25–<30)	5 (12)	9 (6)
Obesity (30 and over)	17 (40)	43 (28)
Median BMI (IQR)	25.1 (23.9–26.6)	23.9 (22.4–26.1)
**Family income per year (baht)**		
Less than 240k	11 (28)	32 (21)
240k–<300k	5 (13)	50 (33)
300k–<400k	7 (18)	29 (19)
400k and over	17 (43)	42 (27)
Unknown	2	1
Median family income per year (IQR)	300,000 (200,000–350,000)	300,000 (240,000–400,000)

Characteristic	Cases (%) (n = 42)	Non-cases (%) (n = 154)
**Province of affiliated unit**		
Ratchaburi	22 (52)	106 (69)
Nakhon Ratchasima	11 (26)	42 (27)
Others	9 (21)	6 (4)
**Main stationed city during the UNMISS**		
Juba	22 (52)	73 (47)
Rumbek	5 (12)	22 (14)
Multiple cities	15 (36)	59 (38)
**Continuous alcohol drinking (yes)**	32 (76)	86 (57)
Unknown	0	2
**Smoking status**		
Current	24 (57)	47 (31)
Former	4 (9.5)	10 (6.5)
Never	14 (33)	96 (63)
Unknown	0	1

Abbreviations: BMI = body mass index; UNMISS = United Nations Mission in South Sudan.
* Seventy-nine individuals were excluded due to unavailability for interviews.
